# Motor mapping–guided resection of a brainstem recurrent pilocytic astrocytoma

**DOI:** 10.3171/2024.10.FOCVID24110

**Published:** 2025-01-01

**Authors:** Xiaopeng Guo, Mitali Bose, Colin Patrick Galvin, Wenya Linda Bi

**Affiliations:** 1 Department of Neurosurgery, Brigham and Women’s Hospital, Harvard Medical School, Boston, Massachusetts;; 2 Department of Neurosurgery, Peking Union Medical College Hospital, Chinese Academy of Medical Sciences and Peking Union Medical College, Beijing, China; and; 3 SpecialtyCare Inc., Neuro Services, Brentwood, Tennessee

**Keywords:** subcortical motor mapping, corticobulbar motor evoked potentials, intraoperative neuromonitoring, corticospinal tract, brainstem, pilocytic astrocytoma

## Abstract

Brainstem tumors are bounded by a compact topography of eloquent tracts, cranial nerves, and nuclei. Reliable intraoperative neuromonitoring aids microneurosurgical technique to optimize safe resection. The authors present a case of motor mapping–guided resection of a recurrent brainstem pilocytic astrocytoma. They demonstrate reliable and continuous responses from the corticospinal tract at the level of the midbrain to the pons with dynamic subcortical motor mapping using a monopolar stimulating suction. Contralateral limb and ipsilateral face muscles can be simultaneously detected and tumor resected until a threshold of less than 1 mA, corresponding to 1 mm or less from the corticospinal tracts.

The video can be found here: https://stream.cadmore.media/r10.3171/2024.10.FOCVID24110

## Transcript

We present a case of motor mapping–guided resection of a brainstem recurrent pilocytic astrocytoma.

### 0:28 Clinical Presentation.

A 25-year-old man who was initially diagnosed with a left ventral midbrain glioma at age 3, treated with biopsy through a retrosigmoid approach and several rounds of chemotherapy, presented with new-onset double vision, corresponding to progressive growth of a multinodular tumor extending from the midbrain to the pons.

### 0:51 Serial Preoperative Imaging.

His original imaging revealed a faintly enhancing expansile tumor abutting the midline of the ventral midbrain. This then developed to a small enhancing nodule of T2-hyperintense and contrast-enhancing lesion medially around 5 years of follow-up, which grew gradually over time. A second enhancing and T2-hyperintense nodule emerged at the lateral inferior aspect of the tumor around 15 years of follow-up, which also steadily grew. At 20 years of follow-up, the tempo of growth accelerated, with acquisition of additional enhancing nodules within the tumor.

### 1:32 Preoperative Tractography.

The tumor originated medial to the cerebral peduncle and subsequently grew laterally and caudally. Therefore, the corticospinal tract (CST) was deflected from the posterolateral to the entire medial aspect of the tumor over time, as supported by the tractography. Of note, on the raw DTI imaging, there is a thin sliver of potential fibers seen in yellow that may course between the dominant posterolateral nodule and a more medial enhancing nodule.

### 2:05 Neurological Examination.

His neurological exam prior to the operation was notable for a mild left abducens palsy and excellent strength throughout, except for trace right-sided weakness from his childhood.

### 2:17 Rationale for the Procedure.

Surgery was offered given the escalating tempo of tumor growth to alleviate symptomatic mass effect,^1^ for cytoreduction, and to obtain tumor tissue for molecular genotyping given the potential for targeted therapies in certain pediatric-type gliomas.

### 2:35 Risks of the Procedure.

Risks of the operation discussed with the patient included worsening weakness, worsening double vision, facial numbness and weakness, stroke, cerebral spinal fluid (CSF) leak, and hydrocephalus.

### 2:49 Positioning.

The patient was positioned supine with trunk elevated, the ipsilateral shoulder bumped up, and the head turned away, with the vertex tilted toward the contralateral shoulder.

### 3:01 Neuromonitoring Modalities.

Intraoperative neuromonitoring was key for this operation and included transcranial motor evoked potentials (MEPs) for monitoring of high-density coverage of the right upper- and lower-extremity muscles and cortically stimulated MEPs for bilateral face, left oculomotor, left abducens, and left masseter muscles. Subcortical motor mapping was set up to allow real-time tracking of the CST for the contralateral extremity muscles and the course of ipsilateral motor cranial nerves from the nuclei to the cisternal segment.^2–5^ Sensory function of the head and body were monitored with somatosensory evoked potentials (SSEPs), left trigeminal SSEPs, and bilateral brainstem auditory evoked potentials. The recording electrode positions for the left oculomotor, left abducens, left masseter, bilateral face muscles and nerves are shown here. The left trigeminal SSEP electrodes are shown in pale blue.

### 4:12 Surgical Video.

I began with extending the prior retrosigmoid craniotomy to allow full exposure of the sigmoid and transverse sinuses for mobilization of the sinus. The dura was opened in a C-shape fashion, beginning with the foramen magnum to release CSF. Extensive adhesions were released until the cyst capsule was exposed medial to the trigeminal nerve. Sharp dissection of the cyst capsule from the undersurface of the trigeminal nerve and the surface of the brainstem was performed to allow for initial decompression of the cyst. This then allowed for handling of the cyst wall to begin dissection against the brainstem. I continued with internal debulking of the tumor using a monopolar stimulating suction throughout the resection to allow for dynamic motor mapping, until low responses were elicited. Here, 1.5 mA of stimulus elicited robust responses from the right hand and smaller responses from the proximal right upper extremity, and down to 0.5 mA of stimulus to see the right hand responses, corresponding to 1 mm or less of distance to the CST. Perforator vessels are carefully preserved. The final rim of tumor was shaved away, until ipsilateral left facial brainstem responses are also elicited on dynamic motor mapping down to 0.8 mA of stimulus, corresponding to less than 1 mm of distance from the facial tract or nucleus. Hemostasis was achieved with Gelfoam saturated with Tisseel fibrin glue. A hypertensive challenge was performed to verify the hemostasis.

### 6:19 Dynamic Subcortical Motor Mapping Guides Extent of Resection.

The dynamic subcortical motor mapping responses throughout the operation is summarized here from top to bottom as time progresses. We began with eliciting right upper-extremity muscle responses at between 7 to 8 mA of stimulus, corresponding to 7 to 8 mm of distance to the CST.^3,4^ This then gradually worked down to 1 and 0.5 mA of stimulus, corresponding to 1 or less than 1 mm of distance to the CST in the right hand responses. Of note, the responses go up and down to reflect the 3D contour of resection with the surrounding CST. The same subcortical mapping also reveals ipsilateral facial responses in the oris and mentalis muscles at various points of the resection down to 0.8 mA of stimulus, corresponding to less than 1 mm from the left facial nucleus or tract.^3,4^ This differs from the robust response seen when the monopolar simulating suction touches the facial nerve, as seen in the responses up above.

### 7:34 Stable Motor Mapping.

At the end of the operation, stable transcranial MEPs are seen along with stable corticobulbar MEPs for the bilateral face and the left masseter muscles. Cortically induced MEPs for the left oculomotor nerve as seen by the superior rectus, the left abducens nerve as seen with the lateral rectus, and the third branch of the trigeminal nerve as revealed by the masseter muscle are also stable at the end of resection. Trigeminal SSEPs remain stable for all three branches of the left trigeminal nerve.

### 8:18 Pathology.

The pathology revealed the classic features of a pilocytic astrocytoma, with a *BRAF-KIAA1549* fusion on genotyping, prompting initiation of tovorafenib therapy.

### 8:32 Clinical Outcome.

The patient experienced resolution of his preoperative double vision and maintained his strength and sensation after the operation. He was begun on tovorafenib at 2 months following the operation.^6^ His postoperative MRI shows near-total resection of the dominant posterior lateral nodule immediately following surgery, with stable to improved disease at 8 months following the operation.

### 9:00 Key Points.

Key points of this case demonstrate the benefit of exposing and mobilizing the transverse and sigmoid sinuses fully, to allow exploration of the supracerebellar infratentorial and lateral cerebellar surfaces without fixed cerebellar retraction. Internal debulking creates space before dissection of the tumor capsule from eloquent tracts. Dynamic motor mapping is able to elicit robust responses from the corticospinal tract at the level of the midbrain and pons. The monopolar stimulating suction allows simultaneous detection of the corticospinal tract for the contralateral limb responses and the course of the motor cranial nerves from the ipsilateral side. Hemostasis is ensured using Gelfoam with Tisseel fibrin glue without electrocauterization to minimize risk of ischemia.

## Disclosures

The authors report no conflict of interest concerning the materials or methods used in this study or the findings specified in this publication.

## Author Contributions

Primary surgeon: Bi. Editing and drafting the video and abstract: all authors. Critically revising the work: Bi, Guo, Bose. Reviewed submitted version of the work: Bi, Guo. Approved the final version of the work on behalf of all authors: Bi. Supervision: Bi. Intraoperative neuromonitoring data: Bose.

## Supplemental Information

### Patient Informed Consent

The necessary patient informed consent was obtained in this study.
